# Facial Expressions in Context: Electrophysiological Correlates of the Emotional Congruency of Facial Expressions and Background Scenes

**DOI:** 10.3389/fpsyg.2017.02175

**Published:** 2017-12-12

**Authors:** Qiang Xu, Yaping Yang, Qun Tan, Lin Zhang

**Affiliations:** Department of Psychology, Ningbo University, Ningbo, China

**Keywords:** emotional congruency effects, facial expression, emotional scene, LPP

## Abstract

Facial expressions can display personal emotions and indicate an individual’s intentions within a social situation. They are extremely important to the social interaction of individuals. Background scenes in which faces are perceived provide important contextual information for facial expression processing. The purpose of this study was to explore the time course of emotional congruency effects in processing faces and scenes simultaneously by recording event-related potentials (ERPs). The behavioral results found that the categorization of facial expression was faster and more accurate when the face was emotionally congruent than incongruent with the emotion displayed by the scene. In ERPs the late positive potential (LPP) amplitudes were modulated by the emotional congruency between faces and scenes. Specifically, happy faces elicited larger LPP amplitudes within positive than within negative scenes and fearful faces within negative scenes elicited larger LPP amplitudes than within positive scenes. The results did not find the scene effects on the P1 and N170 components. These findings indicate that emotional congruency effects could occur in late stages of facial expression processing, reflecting motivated attention allocation.

## Introduction

Facial expressions can display personal emotions and indicate an individual’s intentions within a social situation and, hence, are extremely important for social interaction. A lot of previous studies have explored isolated facial expression processing. However, individuals rarely interact directly with context-less faces. Recently, there has been growing evidence regarding the influence of context on facial expression processing (Aviezer et al., 2008; Barrett et al., 2011; Wieser and Brosch, 2012). It is the primary aim of the present study to contribute to this body of knowledge.

Background visual scenes in which faces are perceived provide important contextual information for facial expression processing. It has been reported that global semantic information about a visual scene could be extracted rapidly because the swift and effective extraction of the gist of visual scenes is based on global cues that are represented by low spatial frequencies in the visual scenes (Bar, 2004; Schyns and Oliva, 2010). So it is possible that background scenes may easily influence facial expression processing due to the rapid extraction of the gist of visual scenes. Actually, some studies began to investigate the effects of background scenes on facial expression processing. For example, behavioral studies found that the recognition of facial expressions was faster and more accurate when faces appeared in emotionally congruent scenes than in emotionally incongruent scenes (Righart and de Gelder, 2008b). Lee et al. (2012) investigated how emotional scenes influenced individual’s facial emotion perception by manipulating the clarity of facial emotions. The results revealed that the more ambiguous the facial emotion was, the more the individual’s perception of facial emotion was influenced by the emotion displayed in the background scene. In addition, using functional magnetic resonance imaging (fMRI), researchers found that the left fusiform gyrus (FG) responded to the emotional congruency between faces and scenes. Specifically, there was increased activity for fearful facial expressions accompanying threatening visual scenes, as opposed to neutral ones. While for neutral faces higher activation of FG was found for neutral scenes than for threatening ones. The results demonstrated that the left FG is involved in processing emotional face–scene congruency effects (Van den Stock et al., 2014).

Based on the excellent temporal resolution of time-locked neural events, event-related potentials (ERPs) have been used to investigate the time course of the integration of emotional information from faces and visual scenes. Righart and de Gelder (2006) used the delayed-response paradigm to examine whether emotional scenes affected the early processing of faces. Although the task was to determine the orientation of the emotional faces (i.e., expressions were task-irrelevant), the study also found that the N170 evoked by faces embedded in the background scenes were modulated by the different emotional scenes. To be specific, the fearful faces embedded in the fearful scenes elicited larger amplitudes than those in the neutral scenes. Subsequently, using an explicit facial expression categorization task (i.e., expressions were task-related), Righart and de Gelder (2008a) found that the N170 was modulated by the congruence of the facial emotion and the emotional scene. In addition, Hietanen and Astikainen (2013) used an emotional priming paradigm to explore the effects of emotional scenes as priming stimuli on the subsequently presented facial expressions. The results showed that the N170 amplitudes in response to sad facial expressions were larger when presented after negative scenes. The emotional congruency effects was also present for happy facial expressions. The N170 amplitudes elicited by facial expressions were modulated by the emotional priming effect. Together, the studies above consistently found that emotional scenes modulated the N170 elicited by emotional faces. The effects showed that the N170 amplitudes were larger when scenes were emotionally congruent rather than incongruent with facial expressions, i.e., emotional congruency effects. As the face-sensitive N170 component, which is a negative ERP component peaking around 140 and 200 ms after stimulus onset at occipito-temporal electrodes, is considered to reflect early perceptual structural encoding of faces (Bentin et al., 1996; Rossion and Jacques, 2008); therefore, it can be concluded that the effects of emotional visual scenes on facial expression processing could occur at the relatively early perceptual coding stage of face processing.

However, the above ERP studies only examined how emotional scenes influenced the early perceptual analysis stages of face processing. What happened at relatively later face processing stages? Could emotional scenes influence face processing at relatively later stages? A recent ERP study used a paradigm similar to previous studies of scene effects for object recognition (Ganis and Kutas, 2003; Demiral et al., 2012). In this paradigm, after presenting a visual scene, a face appeared centrally on the scene. The results showed that when the visual scene appeared before the face-scene compound stimulus, the visual scene influenced facial expression processing, i.e., compared with emotionally congruent face-scene compound stimuli, emotionally incongruent ones elicited a larger fronto-central N2. The effect happened in the post-perceptual stage of facial expression processing and reflected emotional conflict monitoring between emotional visual scenes and facial expressions (Xu et al., 2015). Despite the study demonstrating that the integration of emotional information from faces and visual scenes could arise at the post-perceptual stage when scenes were presented before the face-scene compound stimuli, whether emotional background scenes influence facial expression processing at relatively later face processing stages when faces and scenes were simultaneously processed is still not clear.

In addition, some previous studies provided evidence that verbal descriptions that provide contextual information influenced the perception of facial expressions. For example, in the study of Diéguez-Risco et al. (2013), participants read sentences describing happy or anger-inducing situations and then identified facial expressions that were presented immediately after the sentences. The results showed that the emotional content of verbal descriptions could modulate the amplitudes of the late positive potential (LPP), which demonstrated that the integration of facial expressions with verbal descriptions that provided contextual information occurred at a later stage of face processing. Furthermore, Xu et al. (2016) used an established affective learning procedure to investigate the effects of verbal descriptions when used as context cues on face perception. It showed that faces that were paired with negative social information elicited larger LPPs than faces that were paired with neutral information. Similarly, this study also demonstrated that verbal descriptions used as context cues influenced the face perception at a later stage.

In ERP studies, the LPP component occurs around 400 ms after stimulus onset with a positive peak on centro-parietal sites, and lasts for several hundred milliseconds. Previous studies found that faces with emotional expressions elicited greater LPP amplitudes than neutral faces (e.g., Schupp et al., 2004b). It is considered to reflect the attentional process that is called motivated attention, as it is elicited by emotional stimuli triggering motivational response processes (e.g., avoidance or approach) (Schupp et al., 2004a; Langeslag et al., 2007). Whether emotional congruency between faces and scenes modulates the LPP component that reflected enhanced motivated attention for faces when faces and scenes were simultaneously processed is still unclear.

In the present study we used a paradigm that has been adapted from the Righart and de Gelder study (Righart and de Gelder, 2008a), in which faces and scenes were presented simultaneously, so mental representation of the faces would be constructed in parallel to the construction of the scenes. In this paradigm, after presenting the fixation, the face-scene compound stimulus appeared in the center of the screen. Participants were asked to perform a facial expression categorization task. The emotional information of the faces and scenes was either congruent or incongruent. The purposes of this study were to explore the time course of the integration of facial expressions and emotional information from visual scenes. Apart from early visual ERPs components, i.e., the N170 that reflects early facial structural encoding, as well as the P1 component that peaks positively around 100–130 ms post-stimulus at the parieto-occipital sites and is believed to reflect processing of the low-level physical features of stimuli (Luo et al., 2010), we also investigated the later electrophysiological correlates of facial processing, i.e., the LPP that reflects motivated attention. The present study hypothesizes that emotional scenes could influence facial expression processing at both the early and later stages.

## Materials and Methods

### Participants

As paid volunteers, fifteen healthy college students participated in the experiment (12 females and 3 males, from ages 20 to 23 years, mean = 21.9 years). The participants were all right-handed and had either normal or corrected to normal visual acuity. They also reported no history of mental or neurological diseases. Informed consent was signed by each participant prior to the experiment. The present study was approved by the Ethics Committee of Ningbo University in accordance with the ethical principles of the Declaration of Helsinki.

### Stimuli and Procedure

Thirty-two face pictures (16 females and 16 males) displaying happy and fearful expressions were taken from the native Chinese Facial Affective Picture System (CFAPS) (Gong et al., 2011). Whilst sixteen pictures of positive scenes (e.g., beautiful landscapes, fireworks) and sixteen pictures of negative scenes (e.g., a crashed plane, earthquake ruins) were selected from the International Affective Picture System (IAPS) (Lang et al., 2008) and from the internet in order to match the contents of each of the valence categories. No human or animal faces or bodies appear in any of the scenes. In the pre-test, the collection of 168 emotional scene pictures was rated by a separate group of participants (*N* = 30) on emotional valence and arousal. Ratings were performed on a 9-point scale (valence: from 1 being very unpleasant to 9 being very pleasant and arousal: from 1 being calm to 9 being extremely arousing). According to the results of the ratings, 32 scenes (consisting of 16 positive scenes and 16 negative scenes) were selected. The differences in terms of emotional valence between the positive and negative scenes were significant (positive *M* = 7.20, *SD* = 0.67; negative *M* = 2.38, *SD* = 0.70; *t*(29) = 24.079, *p <* 0.001). There were not significant differences in terms of emotional arousal between the positive and negative scenes (positive *M* = 5.40, *SD* = 1.44; negative *M* = 5.65, *SD* = 1.23; *t*(29) = -0.851, *p =* 0.402). In addition, as control stimuli, scrambled scenes were created by randomizing the position of pixels across the pictures for all 32 intact scenes. The scrambled scenes had the same low-level physical features as the original pictures, but did not contain any semantic information. All compound stimuli in which faces were superimposed onto scene pictures were presented at the center of the screen at a visual angle of 3.81° × 4.40° for facial pictures and 21.83° × 16.37° for scene pictures, respectively. The computer monitor was viewed from a distance of 70 cm.

Participants were seated in a comfortable chair with a straight angle to the center of the computer monitor in a electrically shielded and sound-attenuated room. The experiment consisted of two types of blocks (4 blocks of intact background scenes and 4 blocks of scrambled background scenes), and a total of 8 experimental blocks each containing 64 trials. In each block of intact scenes, background scenes and faces that were superimposed onto scene pictures were combined into four different trial types: happy faces with positive background scenes (congruent), happy faces with negative background scenes (incongruent), fearful faces with negative background scenes (congruent), fearful faces with positive background scenes (incongruent). In each block the four different trial types were presented randomly. Altogether, intact blocks consisted of four conditions, and each condition had 64 trials. Additionally, the pattern of stimulus sequences in the scrambled blocks that were used as control conditions were the same as the intact scene blocks. Scrambled blocks also consisted of four conditions each with 64 trials. The order of intact blocks and scrambled blocks was counterbalanced across participants.

**FIGURE 1:**
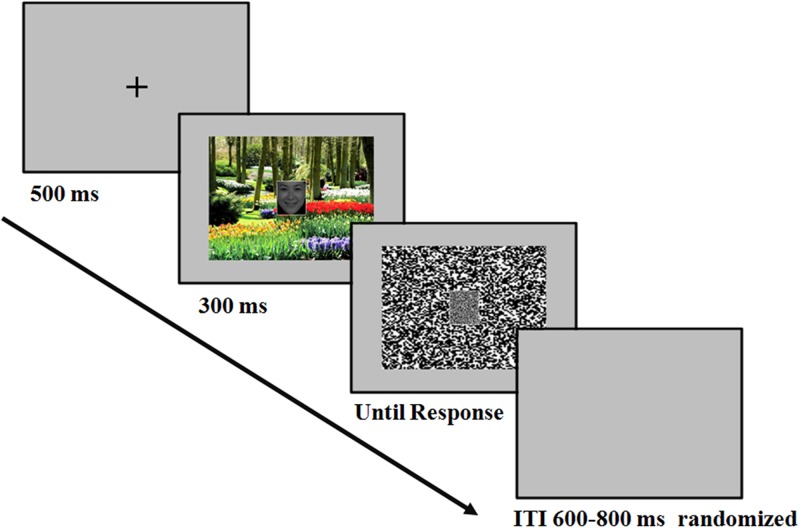
Example of the sequences. After the presentation of the fixation, the face-scene compound stimulus appeared in the center of the screen, which was presented for 300 ms. At stimulus offset, a mask replaced the face-scene compound stimulus until the participant responded. Participants were instructed to perform a facial expression categorization task.

The temporal sequence of the events in a trial was as follows (**Figure 1**): At the beginning of each trial, there was a fixation “+” for 500 ms. Then the face-scene compound stimulus appeared in the center of the screen, which was presented for 300 ms. Then a mosaic mask replaced the face-scene compound stimulus until the participant pressed buttons for response. Participants were informed to concentrate on the face and categorized the facial expression as either happy or fearful by pressing corresponding buttons (labeled with words “happy” and “fearful”). The response button assignments were counterbalanced across participants. The speed and accuracy of the participant’s response were equally emphasized. The inter-trial interval (ITI) varied randomly between 600 and 800 ms. Several practice trials preceded the test trials.

### Electrophysiological Recording and Analysis

The electroencephalogram (EEG) was continuously recorded using an electrode cap with a set of 64 Ag/AgCl electrodes mounted according to the extended international 10–20 system and referenced to the tip of the nose. The horizontal electrooculogram (HEOG) and the vertical electrooculogram (VEOG) were recorded with two pairs of electrodes, one placed on the 10 mm from the lateral canthi, and the other on the left infraorbital and supraorbital areas. Electrode impedance was maintained below 5 kΩ throughout the experiment. The EEG and EOG were amplified and digitalized using a Neuroscan Synamp^2^ Amplifier with a band-pass of 0.05–100 Hz and a sampling rate of 500 Hz.

The EEG was segmented into 1000 ms epochs from 200 ms pre-stimulus to 800 ms post-stimulus, time-locked to face-scene compound stimuli onset and included a 200 ms pre-stimulus baseline. EOG artifacts were corrected offline using a regression-based procedure (Semlitsch et al., 1986). Trials with incorrect responses, or those that were contaminated by bursts of electromyographic activity, amplifier clipping, or peak-to-peak deflection exceeding ± 100 μv were excluded from averaging. The averaged ERPs were digitally filtered with a low-pass filter at 30 Hz (24 dB/octave).

The statistical analysis of ERP data was based on within-subject factorial models in which the peak amplitudes (relative to the pre-stimulus baseline; P1 and N170) and the mean amplitudes (LPP) of original ERP components were dependent variables. The measurement windows were determined by visual inspection of grand-average waveforms, 60–140 ms for P1 and 130–230 ms for N170. The mean amplitudes were measured at the 450–600 ms time windows for LPP. For P1 (PO3, PO4, POz, O1, O2, Oz) and N170 (P7, P8, PO7, PO8, CB1, CB2) components, six electrode sites were analyzed. Based on the LPP scalp distribution, nine electrode sites (F3, F4, Fz, C3, C4, Cz, P3, P4, Pz) were selected for measurement. Peak and mean amplitudes were assessed via repeated measures analysis of variance (ANOVA). If necessary, the degree of freedom were corrected with Greenhouse–Geisser epsilon.

## Results

### Behavioral Performance

Behavioral analyses were performed for response times (RTs) and accuracy rates. For each participant in each condition, incorrect trials or trials with RTs beyond ± 2.5 *SD*s away from the mean were excluded from RT analysis. On average, 8.2% of the trials were removed. Three-way repeated measure ANOVAs were conducted to analyze RTs and accuracy rates with *Facial expression* (fearful, happy), *Scene* (negative, positive), and *Scene picture processing* (intact, scrambled) as within-subject factors.

**FIGURE 2:**
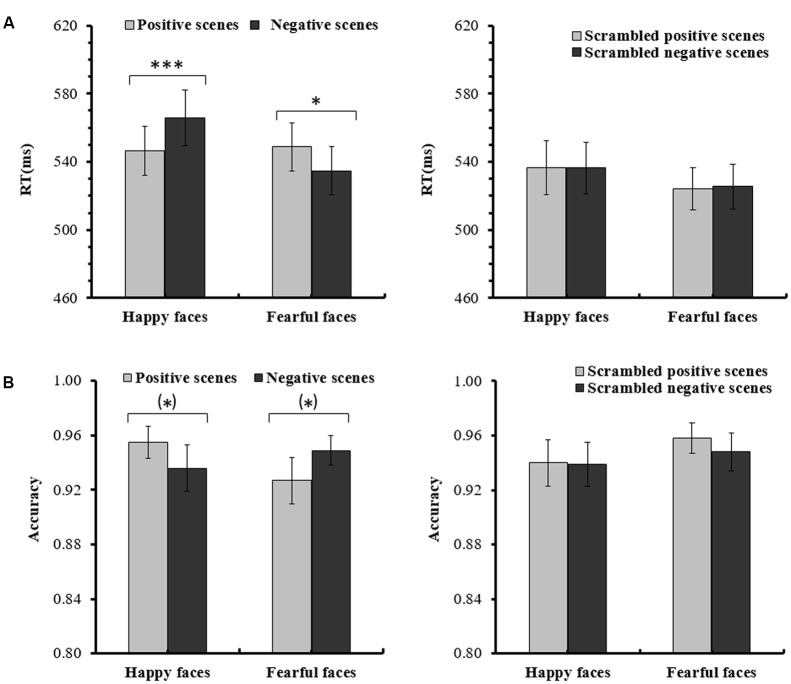
**(A)** Mean (and *SE*) response times for the happy and fearful faces in the different intact or scrambled scenes. **(B)** Mean (and *SE*) accuracy rates for the happy and fearful faces in the different intact or scrambled scenes [^∗∗∗^*p <* 0.001; ^∗^*p <* 0.05; (^∗^)*p <* 0.1].

For the ANOVA analysis of RTs, the main effect of *Scene picture processing* was significant, *F*(1,14) = 18.392, *p <* 0.01, partial η^2^ = 0.568. The interaction of *Facial expression × Scene* was also significant, *F*(1,14) = 22.542, *p <* 0.001, partial η^2^ = 0.617. Importantly, a significant *Facial expression × Scene × Scene picture processing* interaction [*F*(1,14) = 8.898, *p <* 0.05, partial η^2^ = 0.389] was found. Further analysis indicated that for the intact scenes, the RTs for the happy faces in the positive scenes (*M* = 546.60 ms, *SE* = 14.31) were significantly shorter than the RTs for the happy faces in the negative scenes (*M* = 565.68 ms, *SE* = 16.28, *p <* 0.001), and the RTs for the fearful faces in the negative scenes (*M* = 534.57 ms, *SE* = 14.11) were significantly shorter than the RTs for the fearful faces in the positive scenes (*M* = 548.79 ms, *SE* = 14.08, *p =* 0.015). For the scrambled scenes, there were no significant differences for the happy or fearful faces in the different scrambled scenes (*ps* > 0.1) (see **Figure 2** and **Table 1**). None of the other effects for the RTs reached significance (*ps* > 0.05).

**Table 1:** The mean reaction times (RTs in ms) and accuracy rates with (*SE*) for facial expression categorization in the different scenes.

		Happy faces			Fearful faces		
		Positive scenes	Negative scenes	*p*	Negative scenes	Positive scenes	* p*
Intact scenes	RT	546.60 (14.31)	565.68 (16.28)	<0.001	534.57 (14.11)	548.79 (14.08)	0.015
	Accuracy	0.946 (0.012)	0.920 (0.019)	0.090	0.953 (0.014)	0.933 (0.019)	0.072
Scrambled scenes	RT	536.71 (15.80)	536.43 (15.04)	0.956	525.45 (13.21)	524.27 (12.38)	0.708
	Accuracy	0.940 (0.017)	0.939 (0.016)	0.931	0.948 (0.014)	0.958 (0.011)	0.223

For the ANOVA analysis of accuracy rates, there was also a significant interaction of *Facial expression × Scene × Scene picture processing, F*(1,14) = 5.660, *p <* 0.05, partial η^2^ = 0.288. Further analysis indicated that for the intact scenes, the accuracy rates of the happy faces in the positive scenes (*M* = 0.946, *SE* = 0.012) were marginally significantly higher than the happy faces in the negative scenes (*M* = 0.920, *SE* = 0.019, *p =* 0.090), and the differences of the accuracy rates between the fearful faces in the negative scenes (*M* = 0.953, *SE* = 0.014) and the fearful faces in the positive scenes (*M* = 0.933, *SE* = 0.019, *p =* 0.072) were also marginally significant. For the scrambled scenes, no significant differences were found for the happy or fearful faces in the different scrambled scenes (*ps* > 0.1) (see **Figure 2** and **Table 1**). No other main effects and interactions for the accuracy rates were significant (*ps* > 0.05).

### ERPs

#### P1 Components

**FIGURE 3:**
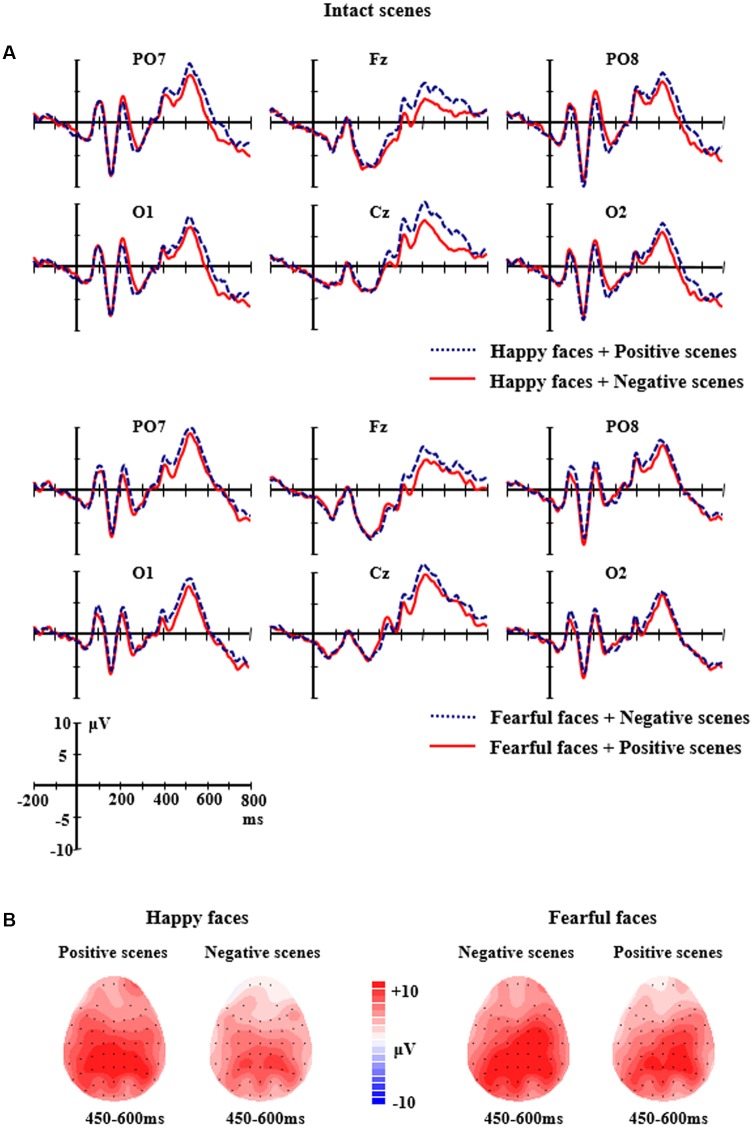
**(A)** The grand-average ERPs elicited by the happy and fearful faces in the different intact scenes at PO7, PO8, O1, O2, Fz, and Cz sites. **(B)** The 2D scalp topographic distribution of the LPP component elicited by the happy and fearful faces in the different intact scenes.

**FIGURE 4:**
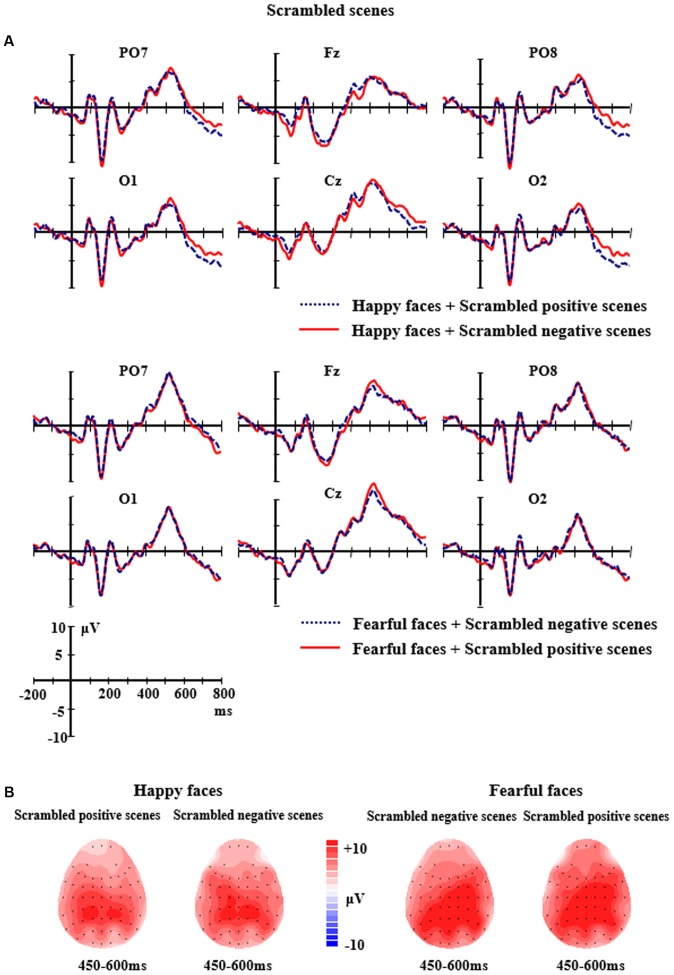
**(A)** The grand-average ERPs elicited by the happy and fearful faces in the different scrambled scenes at PO7, PO8, O1, O2, Fz, and Cz sites. **(B)** The 2D scalp topographic distribution of the LPP component elicited by the happy and fearful faces in the different scrambled scenes.

The grand average ERP waveforms of the relevant components evoked by face stimuli in the intact scene conditions and the scrambled scene conditions are showed by **Figures 3**, **4**. The repeated measure ANOVAs with *Facial expression* (happy, fearful), *Scene* (positive, negative), *Electrodes* (PO3, POz, PO4, O1, Oz, O2) were conducted to analyze the peak amplitudes and latencies of P1.

For the intact scene conditions, the analysis of P1 amplitudes and latencies did not find any main effects or interactions (*ps* > 0.05). For the scrambled scene conditions, the analysis of P1 amplitudes showed a significant main effect of *Electrodes*, *F*(5,10) = 2.909, *p <* 0.05, partial η^2^ = 0.172, reflecting that there was a larger P1 at PO3 site (*M* = 5.052 μV, *SE* = 0.91) than O2 site (*M* = 3.146 μV, *SE* = 0.86). No significant differences of P1 amplitudes were found between other electrodes (*ps* > 0.05). There were no other effects for the P1 amplitudes (*ps* > 0.05). The ANOVA analysis of P1 latencies did not find any significant main effects or interactions (*ps* > 0.05).

#### N170 Components

The peak amplitudes and latencies of N170 were subjected to repeated measure ANOVAs with *Facial expression* (happy, fearful), *Scene* (positive, negative), *Electrodes* (P7, PO7, CB1, P8, PO8, CB2).

For the intact scene conditions, the ANOVA analysis of N170 amplitudes showed the main effect of *Facial expression* was significant, *F*(1,14) = 7.324, *p <* 0.05, partial η^2^ = 0.343, reflecting that the happy faces elicited a larger N170 (*M* = -10.21 μV, *SE* = 1.36) than the fearful faces (*M* = -8.73 μV, *SE* = 1.25). The main effect of *Scene* was also significant, *F*(1,14) = 6.136, *p <* 0.05, partial η^2^ = 0.305, indicating that the faces in positive scenes elicited larger N170 amplitudes (*M* = -9.90 μV, *SE* = 1.31) than the faces in negative scenes (*M* = -9.04 μV, *SE* = 1.28). In addition, a significant main effects of *Electrodes* was found, *F*(5,10) = 6.290, *p <* 0.01, partial η^2^ = 0.310, with a larger N170 at P8 site (*M* = -12.01 μV, *SE* = 1.66) than CB1 site (*M* = -7.91 μV, *SE* = 1.19). No significant differences of N170 amplitudes were found between other electrodes (*ps* > 0.05). The ANOVA of N170 latencies found a significant main effect of *Facial expression, F*(1,14) = 11.903, *p <* 0.01, partial η^2^ = 0.460, with a delayed N170 (*M* = 164.62 ms, *SE* = 2.93) for fearful faces than for happy faces (*M* = 161.06 ms, *SE* = 2.92). Other effects for the amplitudes and latencies of N170 did not reach significance (*ps* > 0.05).

For the scrambled scene conditions, the ANOVA analysis of N170 amplitudes and latencies revealed significant main effects of *Facial expression,* *F*(1,14) = 4.750, *p <* 0.05, partial η^2^ = 0.253 and *F*(1,14) = 22.423, *p <* 0.001, partial η^2^ = 0.616 for N170 amplitudes and latency, respectively, with a larger and earlier N170 (*M* = -11.86 μV, *SE* = 1.50; *M* = 160.97 ms, *SE* = 1.99) for happy faces than for fearful faces (*M* = -10.90 μV, *SE* = 1.50; *M* = 164.55 ms, *SE* = 2.30). Additionally, a significant main effects of *Electrodes* for N170 amplitudes was found, *F*(5,10) = 7.088, *p <* 0.01, partial η^2^ = 0.336, indicating that there were larger N170 amplitudes at P8 site (*M* = -14.73 μV, *SE* = 2.00) than CB1 site (*M* = -9.50 μV, *SE* = 1.39) and CB2 site (*M* = -9.81 μV, *SE* = 1.35). No significant differences of N170 amplitudes were found between other electrodes (*ps* > 0.05). None of the other effects for N170 reached significant levels (*ps* > 0.05).

#### LPP Components

The repeated measure ANOVAs with *Facial expression* (happy, fearful), *Scene* (positive, negative) and *Electrodes* (F3, C3, P3, Fz, Cz, Pz, F4, C4, P4) were conducted to analyze the mean amplitudes of LPP.

For the intact scene conditions, the analysis of LPP amplitudes found a significant two-way interaction of *Facial expression × Scene*, *F*(1,14) = 5.418, *p <* 0.05, partial η^2^ = 0.279. Further analysis showed that the happy faces in the positive scenes elicited larger LPP amplitudes (*M* = 7.33 μV, *SE* = 1.28) than the happy faces in the negative scenes (*M* = 4.98 μV, *SE* = 1.06, *p =* 0.041); the fearful faces in the negative scenes elicited larger LPP amplitudes (*M* = 7.86 μV, *SE* = 1.27) than the fearful faces in the positive scenes (*M* = 6.39 μV, *SE* = 1.18), and the differences were marginally significant (*p =* 0.072). Additionally, there was a two-way significant interaction of *Facial expression × Electrodes*, *F*(8,7) = 6.266, *p <* 0.001, partial η^2^ = 0.309. Further analysis indicated that the LPP amplitudes for the fearful faces were significantly larger than those for the happy faces at the C4 site (*M* = 8.87 μV, *SE* = 1.14 vs. *M* = 6.29 μV, *SE* = 1.20, *p =* 0.011) and the F4 site (*M* = 5.89 μV, *SE* = 1.18 vs. *M* = 3.71 μV, *SE* = 1.03, *p =* 0.039); but the significant differences did not exist at other sites (*ps* > 0.05) (see **Figure 3**).

For the scrambled scene conditions, the analysis of LPP amplitudes showed the main effect of *Electrodes* was significant, *F*(8,7) = 3.872, *p <* 0.05, partial η^2^ = 0.217, followed by a significant two-way interaction of *Facial expression × Electrodes, F*(8,7) = 8.216, *p <* 0.001, partial η^2^ = 0.370. Further analysis indicated that the fearful faces elicited larger LPP amplitudes than the happy faces at the P3, Cz, F4 and C4 sites (P3: *M* = 9.37 μV, *SE* = 1.81 vs. *M* = 7.75 μV, *SE* = 1.67, *p =* 0.038; Cz: *M* = 9.60 μV, *SE* = 1.62 vs. *M* = 7.67 μV, *SE* = 1.42, *p =* 0.031; F4: *M* = 6.85 μV, *SE* = 1.31 vs. *M* = 4.30 μV, *SE* = 1.15, *p =* 0.012; C4: *M* = 9.86 μV, *SE* = 1.50 vs. *M* = 6.44 μV, *SE* = 1.40, *p =* 0.003). None of the other significant effects for the amplitudes of LPP were found (*ps* > 0.05) (see **Figure 4**).

## Discussion

To investigate the time course of the emotional congruency effects between faces and scenes when they were simultaneously processed, the present study used a paradigm that, after presenting the fixation, the face-scene compound stimulus appeared in the center of the screen. Participants performed a facial expression categorization task. The emotional information from the faces and scenes were either congruent or incongruent. We found significant emotional congruency effects in behavioral and ERP results. The behavioral results revealed that the categorization of facial expression was faster and more accurate when the face was emotionally congruent than incongruent with the scene. Specifically, the RTs of the happy faces in the positive scenes were significantly shorter than those in the negative scenes; and the RTs of the fearful faces in the negative scenes were significantly shorter than those in the positive scenes. Additionally, the accuracy rates of the happy faces in the positive scenes were marginally significantly higher than those in the negative scenes, and the differences of the accuracy rates between the fearful faces in the negative scenes and those in the positive scenes were also marginally significant. The ERP results showed that the happy faces in the positive scenes elicited significantly larger LPP amplitudes than those in the negative scenes; the fearful faces in the negative scenes elicited marginally significantly larger LPP amplitudes than in the positive scenes. The results did not find the interactions of *Facial expression × Scene* on the amplitudes and latencies of the P1 and N170 components. The results reflected that the emotional background scenes influenced facial expression processing at later face processing stages when the faces and scenes were simultaneously processed. Additionally, the emotional congruency effects were not found in the scrambled scene conditions. As a result it was possible to determine that the emotional congruency effects did not come as a result of the presence of the low-level physical features of the background scenes.

Consistent with the previous studies, the behavioral results of the present study indicated that the contextual information affected the recognition of facial expressions (Carroll and Russell, 1996; Righart and de Gelder, 2008a,b). Specifically, the recognition of facial expression was faster and more accurate when the facial expression was emotionally congruent rather than incongruent with background scene, i.e., emotional congruency effects. Similarly, Righart and de Gelder (2008a) found that the recognition speed of happy faces in happy scenes was faster than in fearful scenes, but there was no difference in the recognition speed of fearful faces in either of the scenes. Our results found emotional congruency effects for both happy and fearful facial expressions. Although the facial expression categorization task in the present study was relatively simple, the participants had higher accuracy rates in each experimental condition, the results still found that facial expression recognition was more accurate for congruent face-scene compound stimuli than incongruent ones. The results of the present study demonstrated intense emotional congruency effects. Moreover, it was noteworthy that another behavioral study by Righart and de Gelder (2008b) found that the scene effects in facial expression processing still existed even with an increased experimental task load. Indeed, there were some behavioral studies that began to characterize the cognitive nature of the underlying process of face-context integration. For example, using body language as a context cue, Aviezer et al. (2011) investigated the automaticity of emotional face-context integration. The results suggested that facial expressions and their body posture backgrounds were integrated in an uncontrollable, unintentional and relatively effortless manner. As there are essential differences between the integration mechanism of face and scene and the integration mechanism of face and body, whether it is a relatively automatic or controlled process for face-scene integration awaits investigation in further studies.

In our ERP results, the emotional congruency effects between faces and scenes modulated the LPP amplitudes: the LPP amplitudes elicited by happy faces in the positive scenes were larger than those in the negative scenes; and the LPP amplitudes elicited by the fearful faces in the negative scenes were larger than those in the positive scenes, that is, the LPP amplitudes were larger for the congruent emotional conditions when compared to the incongruent conditions. Our results reflected that emotional congruency effects could happen at later stages of face processing. Previous studies demonstrated that compared with neutral stimuli, emotional stimuli evoked larger LPP amplitudes (Schupp et al., 2003, 2004a; Pollatos et al., 2005). The enlarged LPP amplitude that was sensitive to emotional arousal rather than to emotional valence was related to the intensity of emotional stimuli (Cuthbert et al., 2000; Schupp et al., 2000). It is further noted that LPP amplitudes reflect motivated attention which is evoked by stimuli triggering motivational response processes (e.g., avoidance or approach). The enlarged LPP amplitude in response to emotional stimuli indicated the increased motivation as well as the increased attention resources that were allocated in response to the stimuli (Schupp et al., 2004a; Langeslag et al., 2007). For example, Schupp et al. (2004b) found that angry faces elicited larger LPP amplitudes than other facial expressions, indicating that the more motivational attention was allocated to the stimuli that conveyed threatening information, which is important for an individual’s survival. Langeslag et al. (2007) found the LPP amplitudes were larger for the faces of the beloved as opposed to the faces of friends or the faces of attractive strangers. The results implied that more motivated attention was allocated to the faces of the beloved. Our study found that the LPP amplitudes were larger for the congruent emotional conditions (e.g., happy faces in positive scenes) when compared with the incongruent conditions (e.g., happy faces in negative scenes) at frontal, central and parietal sites. The results reflected that the cortical neural response was activated with greater intensity by the emotional faces embedded in the emotionally congruent scenes. And more motivated attention was allocated to the emotional faces embedded in the emotionally congruent scenes. The results implied that these emotional congruency effects were accompanied by increased motivated attention dedicated to the emotionally congruent condition. The effects occurred at the later stage of facial expression processing.

Some ERP studies demonstrated that background scenes influenced facial expression processing during the early perceptual analysis stages of face processing (Righart and de Gelder, 2006, 2008a). These studies found the N170 was larger for some emotionally congruent conditions than incongruent conditions. However, our results did not find the emotional scenes modulated the N170 amplitudes elicited by the facial expressions. The different results between the present study and the Righart and de Gelder (2006, 2008a) studies might be due to the differences of the experimental stimuli and of the paradigms. Righart and de Gelder (2006, 2008a) used specific emotional category scenes (e.g., fearful scenes) as context cues, while the present study used different emotional valence scenes (i.e., positive vs. negative scenes). Compared with the different emotional valence scenes, the specific emotional category scenes might have a close semantic relationship with the emotionally congruent faces (Righart and de Gelder, 2006, 2008a). In this situation their results showed that emotional scenes could affect the processes of facial expression at the early stages of face processing. Nevertheless, using verbal descriptions as a context cue, Xu et al. (2016) found that there are no context effects for the N170 component, which was consistent with our findings. It seems that the features of context stimuli might play an important role in the studies regarding the context effects of face processing. Considering the features of scenes and the semantic relationship between the faces and the scenes, future studies should consider using specific emotional category scenes to investigate how facial expression processing is affected by visual scenes. Furthermore, from the ERP results, the Righart and de Gelder (2006, 2008a) studies found emotional congruency effects for fearful faces. But our results demonstrated emotional congruency effects for both happy and fearful facial expressions, especially for happy ones. The reason for the differences might be that neutral emotional stimuli were also used in their studies, but the present study used stimuli with clear emotional valences.

In the present study, scrambled scenes were used as stimuli for the control condition in order to exclude effects caused by the low-level physical features of the scenes. Scrambled scene pictures may have also been ideal control stimuli in this experimental situation due to the same low-level physical features as the original intact ones, but they do not contain any semantic information. Our findings did not show any interactions from faces and scenes in the scrambled scene conditions, indicating that the emotional congruency effects only occurred in the intact scene conditions. Before concluding, it would be worthwhile to reiterate some of the limitations of the present study. Firstly, different emotional valence scenes (i.e., positive vs. negative scenes) were used as context backgrounds. We mainly investigated the integration of emotional information obtained from faces and scenes. Some researchers believe that semantic information is critical for scene identification, which is important for the emotional response to visual scenes (De Cesarei and Codispoti, 2011). Meaning future studies should explore whether the semantic information in background scenes has an effect on the emotional congruency effects between the faces and the scenes. Secondly, some researchers found gender differences in response to emotional faces that were presented in subthreshold durations (Lee et al., 2017). In the present study unbalanced gender groups (12 females and 3 males) were recruited. Further studies will balance gender groups to explore the emotional congruency effects, and it is also worth investigating gender differences in facial expression processing with background scenes. Thirdly, using verbal descriptions as context cues, Schwarz et al. (2013) found that the emotional and self-reference aspects of contextual information influenced face processing, which was further modulated by individual social anxiety trait. The results suggested individual personality traits should be considered as a modulating factor when exploring context effects on facial emotional processing.

In addition, the present study investigated the time course of the emotional congruency effects between faces and scenes when they were simultaneously processed for normal participants. Therefore, the study laid the foundation for further exploring the specific mechanism of the scene effects of facial expression processing in special individuals. For example, some studies demonstrated that Huntington’s disease mutation-carriers showed deficient explicit recognition of isolated facial expressions (Gray et al., 1997). However, Aviezer et al. (2009) found that the context affected facial expression recognition for both the Huntington’s disease mutation-carriers and the normal participants. Additionally, from the view of context effects, this research also provides a new understanding of the cognitive deficits of prosopagnosia. Aviezer et al. (2012) investigated body context effects of facial expression recognition for a prosopagnosic patient. The study found that the patient could not integrate facial emotion information and body context emotion information very well. Further researches should explore the influence of background scenes on facial expression processing for special individuals as it could play an important role in the recovery of social function in clinical populations.

## Conclusion

The present results emphasize that emotional scenes, when used as a context factor, influenced facial expression processing. Specifically, facial expression processing was enhanced when the scene was emotionally congruent as opposed to incongruent with the face, i.e., emotional congruency effects. It appears as though the effects could occur during later stages of facial expression processing, and may reflect motivated attention allocation.

## Author Contributions

QX conceived and designed the work that led to the submission as well as analyzed data and reviewed the manuscript critically. YY performed the experiment, analyzed data, and reviewed the manuscript critically. QT advised on the study and reviewed the manuscript. LZ advised on the study and revised the manuscript.

## Conflict of Interest Statement

The authors declare that the research was conducted in the absence of any commercial or financial relationships that could be construed as a potential conflict of interest.
